# Do Right-Handed Monkeys Use the Right Cheek Pouch before the Left?

**DOI:** 10.1371/journal.pone.0097971

**Published:** 2014-05-20

**Authors:** Madhur Mangalam, Nisarg Desai, Mewa Singh

**Affiliations:** 1 Biopsychology Laboratory, University of Mysore, Mysore, India; 2 Indian Institute of Science Education and Research Pune, Pune, India; 3 Evolutionary and Organismal Biology Unit, Jawaharlal Nehru Centre for Advanced Scientific Research, Bangalore, India; University of New England, Australia, Australia

## Abstract

There can be several factors that are likely to have played a role in the evolution of hand preference in humans and non-human primates, which the existing theories do not consider. There exists a possibility that hand preference in non-human primates evolved from the pre-existing lateralities in more elementary brain functions and behavior, or alternatively, the two coevolved. A basic example can be a hand-mouth command system that could have evolved in the context of ingestion. In the present study, we examined the relationship between lateralities in prehension and mastication processes, that is, hand and cheek pouch usage, in free-ranging bonnet macaques, *Macaca radiata*. The macaques preferentially used one hand–the ‘preferred’ hand, to pick up the bananas lying on the ground. Lateralities in hand and cheek pouch usage (for both filling and emptying) were positively related with each other, that is, the macaques used the cheek pouch corresponding to the preferred hand predominantly and before the other. Moreover, when the macaques used the non-preferred hand to pick up the bananas, the frequency of contralateral cheek pouch usage was higher than the frequency of ipsilateral cheek pouch usage, that is, the combined structure of hand, mouth, and food did not influence the relationship between laterality in hand usage and laterality in cheek pouch usage. These findings demonstrate laterality in a relatively more involuntary function than those explored previously in any non-human primate species (e.g., facial expressions and manual gestures).

## Introduction

The population-level right-handedness in humans (approximately 90% humans preferentially use the right hand to perform complex manual actions [1], [2]) raises questions about the evolutionary origins of hand preference, in humans as well as in their phylogenetic relatives, the non-human primates [3]. Hand preference in non-human primates has been hypothesized to have had evolved owing to functional and morphological adaptations to feeding in arboreal contexts and to be a precursor of the population-level right-handedness in humans [4]–[6]. Thus, understanding behavioral lateralities in non-human primates is an important first step towards understanding behavioral lateralities in humans. There are two major existing theories that attempt to explain the evolutionary origins of hand preference in non-human primates, namely the task complexity theory [7] and the postural origins theory [4]. The postural origins theory proposes that initially the left hand became specialized for visually guided movements, and the right hand became specialized for postural support. Subsequently, in non-human primate species that adopted a relatively more terrestrial lifestyle, the right hand became more specialized for physical manipulation than for postural support, owing to (a) the development of the opposable thumb and (b) the decreasing demands on the right hand to support vertical posture. However, the postural origins theory fails to describe why initially the left-hand (and not the right hand) became specialized for visually guided reaching, and more importantly, how a population-level right-handedness evolved during the transition from monkeys to apes to humans [8]. Acknowledging that hand preference is likely to be task and situation dependent, the task complexity theory advocates that the cognitively more demanding manual actions that are practiced rarely (e.g., complex and/or bimanual food reaching) would elicit stronger hand preference than the cognitively less demanding actions that are practiced frequently (e.g., unimanual food reaching) [7]. However, the task complexity theory lacks an a priori description of a cognitively demanding manual task and, therefore, remains largely contextual. Also, there can be several other factors that are likely to have played a role in the evolution of hand preference in humans and non-human primates, which are beyond the scope of these two theories.

There exists a possibility that hand preference in non-human primates evolved from the pre-existing lateralities in more elementary brain functions and behavior, or alternatively, the two coevolved. For example, common marmosets, *Callithrix jacchus* have been reported to generally display a larger left hemi-mouth while expressing fear and a larger right hemi-mouth while making social contact calls [9]. Observations from the studies on primate premotor cortex, particularly, on the mirror neuron system, substantiate the hypothesis that initially a dual hand-mouth command system could have evolved in the context of ingestion, which then could have developed into a common platform for manual and vocal communication [10]–[12], i.e., the motor-action patterns that were initially associated with feeding involving both hands and mouth, developed into coordinated manual and vocal gestures. Chimpanzees, *Pan troglodyte* have been reported to preferentially use the right hand in gestural communication, and communicate so more efficiently while vocalizing simultaneously [13].

These observations indicate a strong evolutionary link between laterality in hand usage and laterality in various other elementary brain functions and behavior, describing which can potentially help identifying some general principle(s) underlying behavioral lateralization.

In the present study, we examined the relationship between laterality in the prehension and mastication processes in free-ranging bonnet macaques, *Macaca radiata*. In a previous study on hand usage in bonnet macaques [14], we reported that the macaques preferentially used one hand–the ‘preferred’ hand, to maneuver in three-dimensional space, and the other hand–the ‘non-preferred’ hand, to obtain support. This kind of division of labor is likely to be a more general principle underlying asymmetries in brain functions and behavior with left and right symmetrical components; considering this possibility, we hypothesized that there would be an analogous division of labor in motor-action patterns associated with cheek pouch usage. Bonnet macaques have cheek pouches, which are located in the thickness of the flange on either sides of the mouth within the oral cavity and allow rapid collection and temporary storage/transportation of food. We expected that the macaques would use the cheek pouch corresponding to the preferred hand predominantly and before the other; also, we expected that laterality in hand usage and laterality in cheek pouch usage would be positively related with each other.

## Methods

### Subjects and Study Site

We conducted semi-manipulative experiments on free-ranging bonnet macaques living close to the Chamundeshwari Temple on top of the Chamundi Hills, Mysore, India (2°14′41″N 76°40′55″E). We studied the hand- and cheek-pouch-usage patterns of 14 macaques: 3 adult males, 3 juvenile males, 7 adult females, and 1 juvenile female. Our experiments were completely non-invasive; we placed bananas on the ground within ∼3 m of the macaque and observed their corresponding hand and cheek pouch usage from distance. Our research work adhered to the American Society of Primatologists (ASP) “Principles for the Ethical Treatment of Non-Human Primates.” Whereas no authorization from a local authority was required, we conducted the present study as part of a larger study approved by the Institutional Animal Ethics Committee (IAEC) at the University of Mysore.

### Experimental Procedure

We presented the macaques with bananas that were sufficiently large to fill both the cheek pouches and the mouth, whenever both the cheek pouches were empty. Typically, the macaques picked up the bananas, filled one of the cheek pouches (i.e., either left or right) first, then the other; then they emptied one of the cheek pouches and then the other (which does not require the usage of hands). We recorded the hand used by the macaques in the 21 trials to pick up the bananas lying on the ground, and the chronology of filling and emptying both the cheek pouches, whenever they filled both of them (which allowed us to appropriately determine the laterality in emptying the cheek pouches). To ensure that each data point represented an event, we recorded only one observation corresponding to the act of picking up a banana. And to eliminate the factors that may have influenced hand preference, we presented the macaques with the bananas only when both the hands were free, and discarded those observations in which the presence of conspecific(s) could have conditioned the manual actions.

### Statistical Analyses

We determined the z-scores for hand and cheek pouch usage for each of the macaque using the formula: z-score  = [R−(R+L)/2]/√[(R+L)/4] (where ‘R’ and ‘L’ represent the frequency of usage of the right and left hand respectively), and used the obtained z-scores to determine the preferred hand/cheek pouch (z≤1.96: left; −1.96<z<1.96: none; z≥1.96: right).

We determined the direction and strength of lateral bias in hand usage using the formula: handedness index (HI) = (R−L)/(R+L). The obtained HI values ranged from −1 to +1, with positive values indicating a bias towards right-hand use, and negative values indicating a bias towards left-hand use, while the absolute HI values indicating the strength of the bias. Analogously, we determined the direction and strength of lateral bias in cheek pouch usage viz., laterality index (LI).

We used Spearman's rank correlation tests to determine the relationship between HI and LI values. We used a binomial test to compare the frequency of the usage of the two cheek pouches in the observations in which the macaques used the ‘non-preferred hand’ (i.e., the hand used less often) to pick up the bananas (this allowed us to examine whether physical constraints imposed by the combined structural properties of hand, food, and mouth, ergonomically influenced cheek pouch usage).

## Results

**Figure 1:**
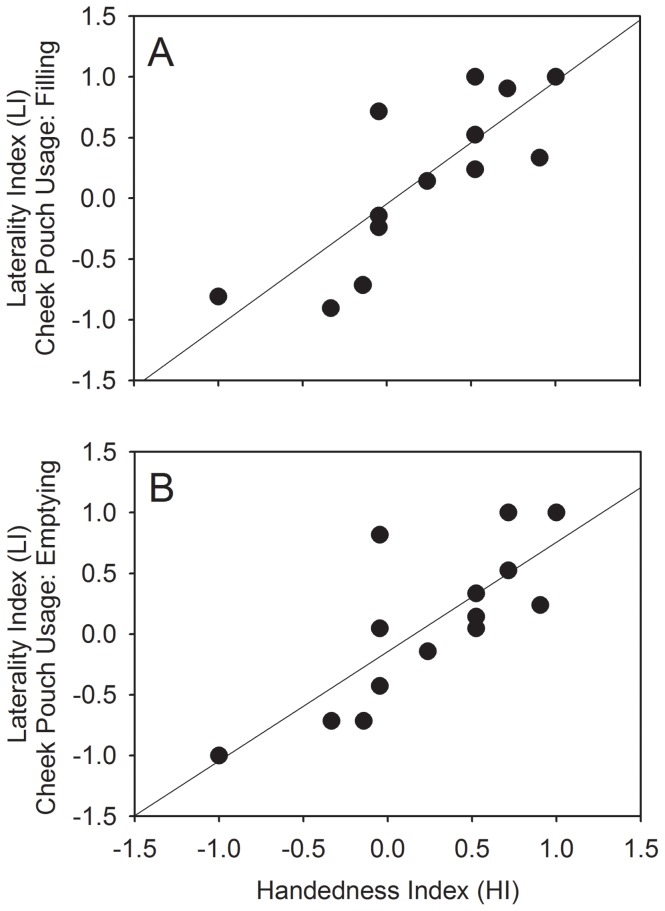
Relationship between Hand and Cheek Pouch Usage among the Macaques (n = 14): while Filling (A) and Emptying (B).

Table 1 describes the hand and cheek pouch usage for the macaques (n = 14). As suggested by the z-scores, only eight macaques preferentially used one hand to pick up the bananas lying on the ground and nine macaques used predominantly and before the other, one of the cheek pouches. There was a positive correlation between the HI and LI values for both filling (Spearman's rank correlation: r_s_ = 0.825, n = 14, p<0.001; Fig. 1A) and emptying (r_s_ = 0.810, n = 14, p<0.001; Fig. 1B) the cheek pouches, and even when the macaques used their non-preferred hand to pick up the bananas, they predominantly used the cheek pouch corresponding to the preferred hand (one-tailed binomial test: 46/76, p = 0.042).

**Table 1:** Hand and Cheek Pouch Usage by the Macaques (n = 14).

Individuals	Hand Usage					Cheek Pouch Usage									
						Filling					Emptying				
	L	R	HI	z-Score	Pref.^a^	L	R	LI	z-Score	Pref.^a^	L	R	LI	z-Score	Pref.^a^
AM1	5	16	0.524	2.400	R	0	21	1.000	4.582	R	7	14	0.333	1.527	N
AM2	21	0	−1.000	−4.582	L	19	2	−0.809	−3.710	L	21	0	−1.000	−4.582	L
AM3	11	10	−0.048	−0.218	N	13	8	−0.238	−1.091	N	15	6	−0.428	−1.964	L
JM1	5	16	0.524	2.400	R	8	13	0.238	1.091	N	9	12	0.143	0.655	N
JM2	11	10	−0.048	−0.218	N	3	18	0.714	3.273	R	2	19	0.809	3.710	R
JM3	3	18	0.714	3.273	R	1	20	0.905	4.146	R	0	21	1.000	4.582	R
AF1	11	10	−0.048	−0.218	N	12	9	−0.143	−0.655	N	10	11	0.048	0.218	N
AF2	5	16	0.524	2.400	R	5	16	0.524	2.400	R	10	11	0.048	0.218	N
AF3	3	18	0.714	3.273	R	1	20	0.905	4.146	R	5	16	0.524	2.400	R
AF4	8	13	0.238	1.091	N	9	12	0.143	0.654	N	12	9	−0.143	−0.655	N
AF5	1	20	0.905	4.146	R	7	14	0.333	1.527	N	8	13	0.238	1.091	N
AF6	12	9	−0.143	−0.655	N	18	3	−0.714	−3.273	L	18	3	−0.714	−3.273	L
AF7	14	7	−0.333	−1.527	N	20	1	−0.905	−4.146	L	18	3	−0.714	−3.273	L
JF1	0	21	1.000	4.582	R	0	21	1.000	4.582	R	0	21	1.000	4.582	R

‘L’ and ‘R’ indicate left and right respectively;

a preferred hand/cheek pouch: z≤1.96: L; −1.96<z<1.96: N (none); z≥1.96: R.

## Discussion

In the present study, we examined the relationship between lateralities (i.e., the direction and strength of preference) in hand and cheek pouch usage in free-ranging bonnet macaques. Lateralities in hand and cheek pouch usage (for both filling and emptying) were positively related with each other, that is, the macaques used the cheek pouch corresponding to the preferred hand predominantly and before the other. Moreover, when the macaques used the non-preferred hand to pick up the bananas, the frequency of contralateral cheek pouch usage was higher than the frequency of ipsilateral cheek pouch usage, that is, the combined structure of hand, mouth, and food did not influence the relationship between laterality in hand usage and laterality in cheek pouch usage. These findings demonstrate laterality in a relatively more involuntary function than those explored previously in any non-human primate species (e.g., facial expressions in rhesus macaques, *Macaca mulatta* [15], [16] and chimpanzees [17], and manual gestures in baboons, *Papio anubis* [18], bonobos, *Pongo pygmaeus* [19], [20], and chimpanzees [13], [21]) (here, ‘relatively more’ refers to the functions that are not entirely involuntary, i.e., they can be voluntary or involuntary to various degrees, but are more involuntary as compared to many other functions).

Asymmetries in elementary brain functions and behavior are turning out to be widespread in both invertebrates and vertebrates [22]–[24]. For example, studies reported that honeybees, *Apis mellifera* [25] and bumble bees, *Bombus terrestris* [26] responded to odors (a first step towards feeding) better when they were trained through the right antenna. Studies on side preference in chewing in humans reported the phenomenon to be comparable to lateralities, for example, in limb usage [27], [28]. And above all, a study on gross preferentially distributed striations on the buccal surfaces of permanent anterior teeth of Neanderthals, *Homo neanderthalensis*, as found in archeological remains, suggested lateralization of the brain functions associated with tool use involving mouth [29]. These examples substantiate the possibility of the ubiquitous nature of lateral asymmetries, extending from very elementary functions to complex motor-action patterns, and across taxa.

Our findings on the relationship between lateralities in hand and cheek pouch usage in bonnet macaques substantiate the possibility of a hand-mouth command system that could have evolved in the context of ingestion, but later could have given rise to hemispheric specializations associated with brain functions and behavior, for example, communication, as has been suggested before [13], [30]. Laterality in cheek pouch usage is likely to be dependent on some endogenous factors that might be related to division of labor or more specifically, the specialization of one cerebral hemisphere for several related/non-related motor-actions. This hypothesis warrants further exploration, more preferably using brain imaging technologies (e.g., magnetic resonance imaging (MRI)) that are now being increasing used in neurophysiological studies on non-human primates (see, for example, studies on capuchins [31]–[33] and chimpanzees [34]. Lateralities in elementary brain functions could have been the precursor of the evolution of lateralities in relatively more voluntary brain functions and behavior. Thus, studies on the evolution of behavioral asymmetries should investigate motor-action patterns beyond hand usage.
